# The G Protein regulators EGL-10 and EAT-16, the G_i_α GOA-1 and the G_q_α EGL-30 modulate the response of the *C. elegans *ASH polymodal nociceptive sensory neurons to repellents

**DOI:** 10.1186/1741-7007-8-138

**Published:** 2010-11-11

**Authors:** Giovanni Esposito, Maria R Amoroso, Carmela Bergamasco, Elia Di Schiavi, Paolo Bazzicalupo

**Affiliations:** 1 Istituto di Genetica e Biofisica A. Buzzati-Traverso, IGB, CNR, Consiglio Nazionale delle Ricerche, Via P. Castellino 111, 80131, Napoli, Italy

## Abstract

**Background:**

Polymodal, nociceptive sensory neurons are key cellular elements of the way animals sense aversive and painful stimuli. In *Caenorhabditis elegans*, the polymodal nociceptive ASH sensory neurons detect aversive stimuli and release glutamate to generate avoidance responses. They are thus useful models for the nociceptive neurons of mammals. While several molecules affecting signal generation and transduction in ASH have been identified, less is known about transmission of the signal from ASH to downstream neurons and about the molecules involved in its modulation.

**Results:**

We discovered that the regulator of G protein signalling (RGS) protein, EGL-10, is required for appropriate avoidance responses to noxious stimuli sensed by ASH. As it does for other behaviours in which it is also involved, *egl-10 *interacts genetically with the G_o/i_α protein GOA-1, the G_q_α protein EGL-30 and the RGS EAT-16. Genetic, behavioural and Ca^2+ ^imaging analyses of ASH neurons in live animals demonstrate that, within ASH, EGL-10 and GOA-1 act downstream of stimulus-evoked signal transduction and of the main transduction channel OSM-9. EGL-30 instead appears to act upstream by regulating Ca^2+ ^transients in response to aversive stimuli. Analysis of the delay in the avoidance response, of the frequency of spontaneous inversions and of the genetic interaction with the diacylglycerol kinase gene, *dgk-1*, indicate that EGL-10 and GOA-1 do not affect signal transduction and neuronal depolarization in response to aversive stimuli but act in ASH to modulate downstream transmission of the signal.

**Conclusions:**

The ASH polymodal nociceptive sensory neurons can be modulated not only in their capacity to detect stimuli but also in the efficiency with which they respond to them. The Gα and RGS molecules studied in this work are conserved in evolution and, for each of them, mammalian orthologs can be identified. The discovery of their role in the modulation of signal transduction and signal transmission of nociceptors may help us to understand how pain is generated and how its control can go astray (such as chronic pain) and may suggest new pain control therapies.

## Background

In *Caenorhabditis elegans*, the two ciliated ASH sensory neurons play a major role in the detection of aversive stimuli and the generation of avoidance responses. The ASH neurons are polymodal in that they are capable of detecting aversive stimuli of different nature (such as water-soluble and volatile chemical repellents, mechanical stress, osmotic shock, pH and heat). Understanding how these neurons function is important as they are useful models of the chemoreceptor and nociceptor neurons present in all animals [1-3]. They can be studied with single-cell resolution in live animals, and their study can take advantage of the powerful molecular and genetic tools available in *C. elegans*. Many molecules acting in ASH and necessary for signalling have been identified: GPCRs (G Protein Coupled Receptors); G proteins, including several sensory neuron-specific Gα subunits; regulators of G protein signalling (RGS); and various types of channels [1,3]. The pathways by which different noxious stimuli signals function have not been completely dissected, but it is well established that, for all the aversive stimuli tested so far, the different pathways converge and use the transient receptor potential vanilloid-related (TRPV) channel protein OSM-9, which is the main sensory transduction channel of ASH neurons [4]. Gating of OSM-9 at the dendritic sensory cilia, where it is localised, triggers the depolarisation of the neuron [5]. The ASH neurons are glutamatergic and signal to the downstream command interneurons (AVA, AVB, AVD and PVC) that control forward and backward movement through motoneurons and muscle [3,6]. Avoidance responses are modulated by environmental and internal cues and by previous experience. Modulation can occur at different points of the underlying neural circuit, including sensory neurons. Various molecules have been identified that are involved in the modulation of the sensitivity of ASH by increasing or decreasing signal transduction and depolarisation [3,5,7-10]. Less is known about the mechanisms and molecules that in ASH modulate the transmission of the signal to the downstream interneurons. Exceptions are *nlp-3*-encoded peptides and their receptor, NPR-17, that mediate serotonin-dependent stimulation of ASH aversive response to dilute octanol [11] and the EGL-3 proconvertase, which appears to modulate ASH transmission but functions in the downstream interneurons and presumably affects ASH through secreted neuropeptides [12].

Here we show that the RGS EGL-10 affects avoidance responses by functioning in ASH and interacting with the G_o/i_α protein GOA-1, the G_q_α protein EGL-30, the RGS EAT-16 and the diacylglycerol kinase DGK-1. EGL-10 does not affect ASH signal generation and transduction but acts, by inhibiting GOA-1 signalling, downstream of TRPV/OSM-9 and of the L-type voltage-gated Ca^2+ ^channel (L-VGCC) EGL-19 to modulate transmission of the signal to downstream neurons. EGL-30 instead appears to act upstream of TRPV/OSM-9 to regulate ASH Ca^2+ ^transients triggered by repellents.

## Results

### Loss of *egl-10 *function affects the response to aversive stimuli

**Figure 1:**
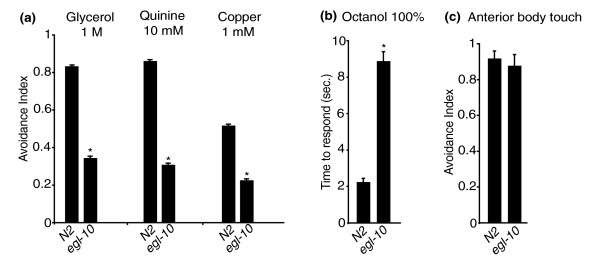
***egl-10 *is involved in avoidance of aversive stimuli**. N2 is the wild-type control, and *egl-10 *is *egl-10*(*md176*). For each genetic background, ≥50 animals were tested in at least three independent assays: in **(a) **and **(b)**, each animal was subjected to three trials; in **(c)**, each animal was subjected to 30 trials. The avoidance index is the number of positive responses divided by the total number of trials. In (B), avoidance is expressed as the time in seconds that the animal took to respond. In all panels, each bar represents the mean ± SEM. *Difference from N2, *P *< 0.01.

*egl-10 *encodes a conserved regulator of G protein signalling (RGS) protein involved in egg-laying and locomotion behaviour, and loss of its function causes defective egg-laying and sluggish movement [13]. To test avoidance, we used the drop test assay [6] and found that animals carrying *md176*, a null allele of *egl-10 *[13], were defective in the response to high osmotic strength, quinine and copper ions with only 25 to 35% of the animals responding within the 3 seconds of the assay (Figure 1a). To further characterize the defect of *egl-10 *mutants, we also used a modification of the assay, the dry drop test (see Methods), and measured the time interval in seconds (delay) between the initial contact of the animal with the aversive stimulus (high osmotic strength) and the beginning of the backward movement. We found that *egl-10 *animals responding to the stimulus within 7 seconds (about 50% of them) took significantly longer than wild-type animals (2.8 ± 0.17 seconds versus 1.2 ± 0.1 seconds) (Table 1). This shows that a significant fraction of *egl-10 *mutant animals eventually respond to the repellent, although they take much longer than wild-type animals. *egl-10 *mutant animals also took much longer than control animals to respond to the volatile repellent octanol (100%; Figure 1b). Thus the avoidance response does not appear to be completely abolished, but is instead strongly downregulated, suggesting a modulatory role for EGL-10 in this behaviour.

**Table 1:** Delay in avoidance response

Genotype	Responding (%)	Response delay in seconds (means ± SEM)	Number of worms	Repellent
N2	88	1.19 ± 0.10	100	
*egl-10*	48*	2.83 ± 0.17*	100	
*egl-10;*p*sra-6::egl-10*	83**	1.90 ± 0.18**	90	1 M glycerol
*dgk-1*	92	1.14 ± 0.08	80	
*egl-10;dgk-1*	78**	1.32 ± 0.09**	80	
				
p*sra-6::TRPV1*	100	2.56 ± 0.16	90	50 μM capsaicin
*egl-10;*p*sra-6::TRPV1*	88°	4.48 ± 0.20***	90	

Dry drop test (see Methods). Responding animals are those that begin backward movement within 7 seconds from encountering the repellent. *Difference from N2, *P *< 0.01; **difference from *egl-10*(*md176*), *P *< 0.01; ***difference from p*sra-6::TRPV1*, *P *< 0.01.

### *egl-10 *interacts with *goa-1*, *egl-30 *and *eat-16*

**Figure 2:**
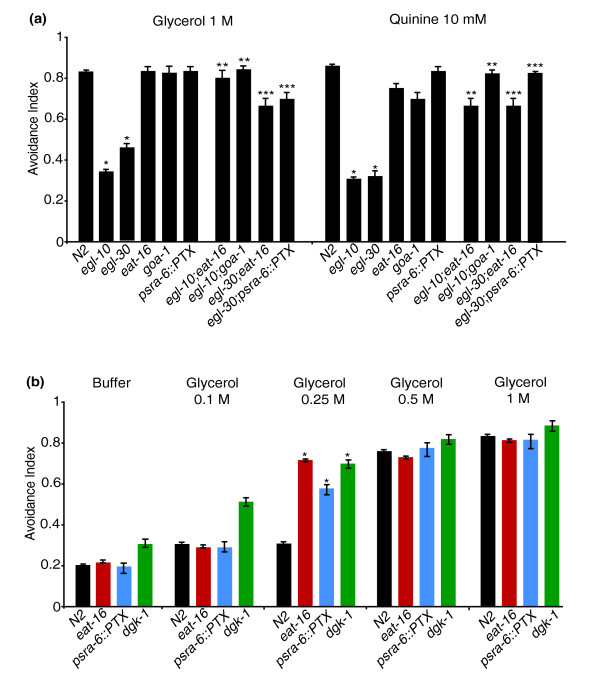
**Avoidance responses of single and double mutants**. N2 is the wild-type control, *egl-10 *is *egl-10*(*md176*), *egl-30 *is *egl-30*(*n686*), *eat-16 *is *eat-16*(*ad702*), *goa-1 *is *goa-1*(*n363*) and *dgk-1 *is *dgk-1*(*nu62*). The transgene p*sra-6*::PTX is described in the text, in the Results section and represents an ASH-specific *goa-1 *loss of function. For each genetic background, ≥50 animals were tested in at least three independent assays, and each animal was subjected to three trials. The avoidance index is the number of positive responses divided by the total number of trials. In all panels, each bar represents the mean ± SEM. In **(b)**, different concentrations of glycerol were used to detect the hypersensitivity of the wild type and of the *eat-16, goa-1 *and *dgk-1 *mutants. *Difference from N2, *P *< 0.01; **difference from *egl-10*(*md176*), *P *< 0.01; ***difference from *egl-30*(*n686*), *P *< 0.01.

Previous work has shown that in *C. elegans*, egg-laying and locomotion, two behaviours affected by *egl-10*, are controlled by two opposing G protein signalling pathways involving the G_o/i_α protein GOA-1 and the G_q_α protein EGL-30. In particular, GOA-1 activity inhibits egg laying and locomotion, whereas EGL-30 has the opposite effect [14]. Genetic and biochemical experiments have shown that the RGS protein EGL-10 is a specific inhibitor of GOA-1 activity and that the RGS protein EAT-16 is a specific inhibitor of EGL-30 [15]. We asked whether the four genes interact in a similar way to control also the response to aversive stimuli. We used the *egl-30*(*n686*) and the *eat-16*(*ad702*) hypomorphic alleles and the *goa-1*(*n363*) null allele and tested the avoidance behaviour of single- and double-mutant animals. Similarly to *egl-10 *mutants, *egl-30*(*n686*) animals also are defective in avoidance responses, while the responses of *goa-1*(*n363*) and *eat-16*(*ad702*) animals were not significantly different from those of wild-type animals (Figure 2a). Similarly to the egg-laying and locomotion defects, the avoidance defects of *egl-10*(*md176*) were completely suppressed by the mutations *eat-16*(*ad702*) or *goa-1*(*n363*) (Figure 2a). In addition, the *eat-16*(*ad702*) mutation also suppressed the defects of *egl-30*(*n686*). With regard to the *goa-1*, *egl-30 *double mutant, as found by other researchers before us [16], these double-mutant animals proved difficult to maintain, became extremely sick and were impossible to be assayed reliably for avoidance. To bypass this problem, we decided to use, instead of the *goa-1*(*n363*) allele, animals in which GOA-1 is specifically inactivated only in ASH and a few other neurons. We used the *sra-6 *promoter to express the catalytic subunit of the pertussis toxin (PTX) (a gift from M. Koelle, Yale University, New Haven, CT, USA) selectively in the ASH neurons. p*sra-6 *is active also in the ASI and PVQ neurons [17], but these neurons are not involved in avoidance. PTX inactivates G_o_α proteins by ADP ribosylation of a conserved cysteine, and, in *C. elegans*, it has been used to specifically inactivate GOA-1 [18]. We found that inactivation of GOA-1 by PTX in the ASH neurons (using a p*sra-6::PTX *transgene) rescues the *egl-30*(*n686*) avoidance phenotype (Figure 2a).

If the two opposing pathways mechanism that regulates egg-laying functions also in avoidance, one would expect *eat-16 *and *goa-1 *mutants to be hypersensitive to aversive stimuli. However, as Figures 2a and 2b show, 1 M glycerol, the high osmotic strength stimulus used in our standard drop test, is too strong a stimulus to allow detection of hypersensitivity. We thus challenged *eat-16*(*ad702*) and p*sra-6::PTX *animals with increasing concentrations of glycerol and found that their response to 0.25 M glycerol is in fact significantly higher than that of wild-type animals (Figure 2b).

*eat-16 *and possibly *goa-1*, mutants have a higher frequency of spontaneous reversals (see paragraph "EGL-10 may function by modulating synaptic transmission of ASH"). Thus, in principle, their observed rescue of the avoidance defects of *egl-10*(*md176*) and *egl-30*(*n686*) (Figure 2a) may simply be the result of the increased frequency of spontaneous reversals in double-mutant animals. However, the response to mock stimulation (drop test with buffer alone without repellent) of all the single- and double-mutant strains used were indistinguishable from those of the wild type, with avoidance indexes ranging between 0.15 and 0.25 (Figure 2b and Additional file 1). This result indicates that, as administered, the drop test assay distinguishes between spontaneous reversal and avoidance response and that the restored avoidance responses of the double-mutant animals are true rescues. Overall the genetic interaction experiments show that the four genes interact in a similar way to affect avoidance, egg laying and locomotion, with GOA-1 acting in an inhibitory pathway negatively modulated by EGL-10 and with EGL-30 acting in a stimulatory pathway negatively modulated by EAT-16.

### EGL-10 functions in the ASH sensory neurons

**Figure 3:**
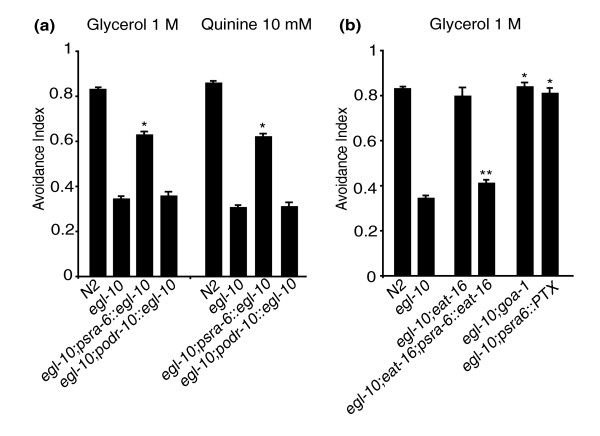
***egl-10*, *goa-1 *and *eat-16 *function in ASH to affect avoidance**. N2 is the wild-type control, *egl-10 *is *egl-10*(*md176*), *eat-16 *is *eat-16*(*ad702*) and *goa-1 *is *goa-1*(*n363*). The transgenes p*sra-6::egl-10*, p*sra-6::eat-16*, p*odr-10::egl-10 *and p*sra-6*::PTX are described in the text, in the Results and in the Methods sections. p*sra-6*::PTX represents an ASH-specific *goa-1 *loss of function. For each genetic background, ≥50 animals were tested in at least three independent assays, and each animal was subjected to three trials. The avoidance index is the number of positive responses divided by the total number of trials. In all panels, each bar represents the mean ± SEM. *Difference from *egl-10*(*md176*), *P *< 0.01; **difference from *egl-10*(*md176*); *eat-16*(*ad702*), *P *< 0.01. For each genetic background, only the results obtained with animals from one transgenic line are represented in the figure. The results from the other lines obtained and tested are reported in Additional file 2.

EGL-10 and its genetic partners are widely expressed in the nervous system [13] and could, in principle, affect avoidance behaviour by acting in sensory neurons or in downstream neurons of the underlying circuit. Aversive stimuli are detected by amphidial sensory neurons with the polymodal nociceptive neuron, ASH, playing the main role in the detection of the stimuli for which *egl-10 *mutants are defective [1,3,17,19-21]. Light anterior body touch, a mechanical stimulus that also triggers an avoidance response, is detected not by ASH but by the mechanoreceptors ALM and AVM [3,22]. The downstream command interneurons to which ASH, ALM and AVM connect, however, are largely overlapping. Avoidance of light anterior body touch is intact in *egl-10 *mutant animals (Figure 1c), suggesting that the avoidance phenotype of *egl-10 *mutants is more likely the result of a defect in the sensory neurons than in the downstream neurons of the circuit, unless other as yet unidentified neurons play a role in these avoidance responses. To test directly whether, for avoidance, the function of *egl-10 *is required in ASH, we introduced in *egl-10 *mutants the wild-type *egl-10 *cDNA and expressed it specifically in the ASH neurons under the *sra-6 *promoter (see paragraph above "*egl-10 *interacts with *goa-1*, *egl-30 *and *eat-16"*). The p*sra-6*::*egl-10 *transgene was sufficient to rescue ASH-mediated responses to quinine and to high osmolarity (Figure 3a). It also rescued the delay time in the response to the high osmotic strength stimulus (Table 1). As expected, the transgene did not rescue the egg-laying phenotype or the sluggish movement (not shown). A control transgene driving expression in the AWA sensory neurons under the *odr-10 *promoter did not rescue the avoidance phenotype (Figure 3a).

To test whether *eat-16 *acts in ASH, we transformed *eat-16*;*egl-10 *double-mutant animals with a transgene in which the *eat-16 *genomic coding sequence was expressed in ASH under the *sra-6 *promoter. In these double-mutant animals, the transgene restored the avoidance defects observed in *egl-10 *single mutants (Figure 3b). To test whether *goa-1 *is acting in ASH to affect avoidance, we used the p*sra-6::PTX *transgene (see paragraph above "*egl-10 *interacts with *goa-1*, *egl-30 *and *eat-16"*). Inactivation of GOA-1 by PTX specifically in the ASH neurons completely rescued the avoidance defects of *egl-30 *(Figure 2a) and of *egl-10 *mutants and resulted in an avoidance phenotype similar to that of *egl-10*;*goa-1 *double mutants (Figure 3b). Taken together, these results indicate that EGL-10, GOA-1 and EAT-16 control avoidance by acting in the ASH sensory neurons.

### EGL-10 acts downstream of the TRPV channel OSM-9

In *egl-10 *mutant animals, the amphidial neurons, including ASH, stain normally with the lipophylic dyes DiI (1,1'-dioctadecyl-3,3,3',3'-tetramethylindocarbocyanine perchlorate) and DiO (3,3'-dioctadecyloxacarbocyanine perchlorate) [23] (not shown), indicating that loss of *egl-10 *function does not cause major structural alterations of these neurons and/or of their cilia and that the avoidance phenotype is more likely due to defects in ASH signal transduction or transmission. Within ASH, EGL-10 could function in the signal transduction upstream of OSM-9 or downstream of it. *C. elegans *does not respond to the chili pepper irritant capsaicin, but animals expressing the rat TRPV1 channel in ASH respond to capsaicin with an escape behaviour similar to the avoidance of the aversive stimuli normally sensed by ASH [4]. Capsaicin directly activates TRPV1 to depolarise ASH and trigger an avoidance response that bypasses the signal transduction upstream of and including OSM-9, but that requires the vesicular glutamate transporter EAT-4 [4]. Thus mutants in genes acting upstream of OSM-9 respond normally to capsaicin, while mutants in genes acting downstream are defective. We used a p*sra-6*::TRPV1 transgene (a gift from C.I. Bargmann, Rockefeller University, New York, NY, USA) that drives the expression of the rat TRPV1 channel in ASH and measured the avoidance response to capsaicin of *egl-10*(*md176*) and wild-type animals transgenic for p*sra-6*::TRPV1. We found that the response of *egl-10*(*md176*);p*sra-6*::TRPV1 animals was significantly delayed compared to controls (4.5 ± 0.20 seconds versus 2.6 ± 0.16 seconds; *P *= 0.000) (Table 1), with a fraction of *egl-10 *mutant animals not responding at all (12%). Thus the avoidance defect of *egl-10 *mutants is not bypassed by the expression of TRPV1, indicating that EGL-10 is required in ASH downstream of the TRPV/OSM-9 channel.

### ASH neurons of *egl-10 *mutants show normal stimulus-evoked Ca^2+ ^transients

**Figure 4:**
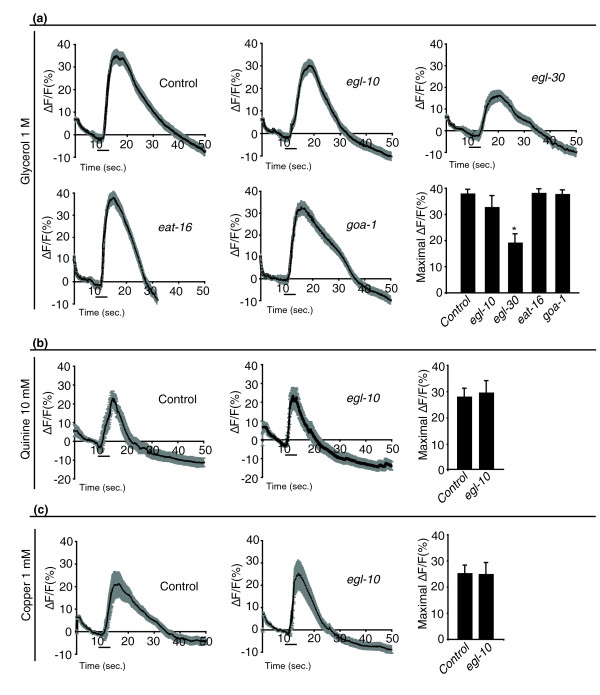
***egl-10, eat-16 *and *goa-1*, but not *egl-30 *mutant animals show normal stimulus-evoked Ca^2+ ^transients in ASH neurons**. Ca^2+ ^transients in ASH neurons. All animals carry the p*sra-6*::G-CaMP transgene driving expression of the G-CaMP Ca^2+ ^sensor in ASH. Genetic background is wild type for control, *egl-10*(*md176*) for *egl-10*, *egl-30*(*n686*) for *egl-30*, *eat-16*(*ad702*) for *eat-16 *and *goa-1*(*n363*) for *goa-1*. ASH Ca^2+ ^transients are reported in response to **(a) **high osmotic strength stimulus, **(b) **quinine and **(c) **copper. For each repellent and for each genotype, at least 20 animals were tested. Three individual imaging trials were recorded for each animal. In the graph panels, the time courses of the change in green fluorescent protein (GFP) fluorescence intensity after stimulus delivery are shown relative to averaged prestimulus (10 seconds) baseline (ΔF/F); the grey bands represent the SEM for each time point (0.2 second). The black horizontal bars indicate the time and duration of the stimulus. In the histogram panels, the means ± SEM of the maximum ΔF/F of all the imaging trials for a given stimulus and genotype are reported. *Difference from control, *P *< 0.01.

It has been shown that aversive stimuli induce Ca^2+ ^transients in ASH that require stimulus-specific signal transduction components as well as the TRPV channel OSM-9 at the tip of the sensory cilia [5]. Experimentally observable Ca^2+ ^transients in the ASH cell body require, in addition, the L-type voltage-gated calcium channel (L-VGCC) EGL-19 to conduct the depolarization from the cilia to the cell soma [5]. We imaged Ca^2+ ^fluxes in ASH cell bodies *in vivo *[24] in animals that express the genetically encoded Ca^2+ ^sensor, G-CaMP [25], under the *sra-6 *promoter (a gift of J. Nakai, Saitama, Japan, and of C.I. Bargmann, Rockefeller University, New York, NY, USA). We found that the slope, duration and intensity of Ca^2+ ^transients evoked by high osmolarity, quinine and copper in *egl-10 *mutants were indistinguishable from those of wild-type animals (Figures 4a, b and 4c). We also measured ASH Ca^2+ ^transients in response to the high osmotic strength stimulus in *eat-16*, in *goa-1 *and in *egl-30 *mutant animals. Also in these mutants, the Ca^2+ ^transients were like those of wild-type animals, except for *egl-30 *mutants, in which the intensity was reduced (to about 60%) but not abolished (Figure 4a). Avoidance defects can result from reduced ASH signalling but also from abnormally high signalling, as occurs in *rgs-3 *mutants [26]. Ca^2+ ^sensors such as G-CaMP or Cameleon can function as Ca^2+ ^sponges, and it has been shown that the expression of Cameleon in ASH restores normal behavioural responses and Ca^2+ ^transients in *rgs-3 *mutants [26]. To find out whether this was occurring also in our case, we tested the avoidance behaviour of *egl-10*, *eat-16*, *goa-1 *and *egl-30 *mutants carrying the p*sra-6*::G-CaMP transgene and found that the transgene did not cause changes in their avoidance responses (not shown). These results directly demonstrate that EGL-10, EAT-16 and GOA-1 are not required in ASH to generate Ca^2+ ^transients in response to aversive stimuli. The reduction of *egl-30 *function instead reduces the intensity of ASH Ca^2+ ^transients in response to high osmolarity, suggesting that this Gα protein functions by modulating stimulus-evoked signalling upstream of OSM-9.

### EGL-10 may function by modulating synaptic transmission of ASH

Calcium imaging of ASH indicates that, in *egl-10 *mutants, primary stimulus-evoked signalling is not compromised and results in normal depolarisation and Ca^2+ ^transients that reach the cell body. This suggests that the avoidance phenotype of *egl-10 *mutants derives from defects in the transmission of the signal to downstream neurons in the circuit. This hypothesis is also consistent with previous data indicating that EGL-10 localises at the sites of chemical synapses [13] and that, in animals overexpressing *egl-10*, neurotransmitter release at neuromuscular junctions is increased [16].

Reduction of synaptic transmission between ASH and the command interneurons results in a delay of the withdrawal response to an aversive stimulus, rather than in a complete absence of the response [12]. Thus the efficiency of synaptic transmission can be measured by the delay in the withdrawal response, and our finding of such delay in the response to the osmotic strength stimulus of *egl-10 *mutants (Table 1) is consistent with the hypothesis that *egl-10 *controls neurotransmitter release of ASH.

Previous work has shown that the opposite effects of *egl-30 *and *goa-1 *on egg laying and locomotion are exerted through opposing effects on motoneuron transmitter release [16,27,28]. The EGL-30 G_q_α and the EGL-8 phospholipase Cβ stimulate production of presynaptic diacylglycerol (DAG), which facilitates acetylcholine release at neuromuscular junctions by regulating synaptic vesicle exocytosis [27]. The G_o_α, GOA-1 and the DAG kinase, DGK-1, inhibit acetylcholine release, decreasing DAG levels at nerve terminals: GOA-1 by decreasing DAG production and DGK-1 by converting DAG to phosphatidic acid [28]. ASH is a sensory neuron, and it signals to the command interneurons via glutamate and not acetylcholine. We asked whether EGL-10 might modulate neurotransmitter release also in ASH through a mechanism similar to that acting at neuromuscular junctions. EGL-10 could positively regulate glutamate release by inhibiting GOA-1 signalling and thus increase DAG levels. Loss of *egl-10 *function would reduce DAG at the presynaptic terminal, resulting in delayed avoidance responses, while *dgk-1 *loss of function should increase DAG levels. *nu62 *is a loss of function allele of *dgk-1 *[27], and we could show that *dgk-1*(*nu62*) animals are hypersensitive to the high osmotic strength stimulus (Figure 2b). We measured the delay in the response to the high osmotic strength stimulus of *dgk-1*(*nu62*)*;egl-10*(*md176*) double-mutant animals. Consistent with the hypothesis, we found an almost complete rescue of the avoidance phenotype of *egl-10 *single mutants with regard to both the number of responding animals and the delay in the response time (Table 1).

**Figure 5:**
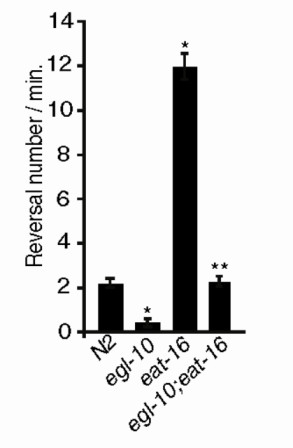
***egl-10 *and *eat-16 *have opposite effects on the frequency of spontaneous reversals**. N2 is the wild-type control, *egl-10 *is *egl-10*(*md176*) and *eat-16 *is *eat-16*(*ad702*). For each genetic background, ≥50 animals were tested in at least three independent assays. Numbers of spontaneous inversions per minute are represented. Each bar represents the mean ± SEM. *Difference from N2, *P *< 0.01; **difference from *egl-10*(*md176*), *P *< 0.01.

Even in the absence of aversive stimuli, worms spontaneously interrupt their forward movement with brief backward reversals. Glutamatergic input to the command interneurons, provided by ASH and by other sensory neurons, has been shown to bias backward movement [29]. The frequency of spontaneous reversals during locomotion has thus been used in behavioural assays to test the strength of glutamatergic synaptic transmission [30,31] and has been shown to be regulated presynaptically by the basal level of activation and firing of ASH and other sensory neurons and postsynaptically by the concentration of glutamate receptors [29,30,32,33]. We measured the frequency of spontaneous reversals of wild-type, *egl-10*(*md176*) and *eat-16*(*ad702*) animals as well as that of *egl-10*(*md176*);*eat-16*(*ad702*) double-mutant animals. Compared to wild type, the frequency of reversals per minute was lower in *egl-10 *and higher in *eat-16 *mutant animals, while *egl-10;eat-16 *double mutants had the same number of reversals as wild type (Figure 5). These results are again consistent with the interpretation that EGL-10 and GOA-1 affect avoidance behaviour by modulating glutamatergic transmission presynaptically in the ASH neurons.

## Discussion

We have shown that the RGS protein EGL-10 and its partners, the G_0/i_α GOA-1, the G_q_α EGL-30 and the RGS EAT-16 interact genetically in the ASH sensory neuron to modulate avoidance responses of *C. elegans *to aversive stimuli. Thus the same set of interacting signalling proteins modulates behaviour at the output (neuromuscular junctions) and input (sensory neurons) ends of the neural circuit underlying avoidance. We show that, in ASH, EGL-10 does not affect primary signal transduction but acts downstream of the main signal transducer channel OSM-9 and of the propagation of stimulus-evoked Ca^2+ ^transients to the cell body. The delay in the avoidance response of *egl-10 *mutants, the frequency of spontaneous, non-stimulus-evoked reversals of locomotion and the genetic interaction with the DAG kinase gene, *dgk-1*, suggest that EGL-10 contributes to the regulation of neurotransmitter release at the ASH synapses. The results of the genetic interactions and Ca^2+ ^imaging experiments on *goa-1 *mutants indicate that GOA-1 also acts, in ASH, downstream of OSM-9 and that it interacts with EGL-10 in a fashion similar to that in which these two proteins control acetylcholine release at the neuromuscular junction. The Ca^2+ ^imaging results on *egl-30 *mutant animals show that, in ASH, this G_q_α protein influences Ca^2+ ^transients and depolarisation in response to high osmolarity, indicating a modulatory role for this protein in ASH signal transduction. With regard to EAT-16, our results show that this RGS is required in ASH to modulate avoidance responses with effects opposite to those of EGL-30.

**Figure 6:**
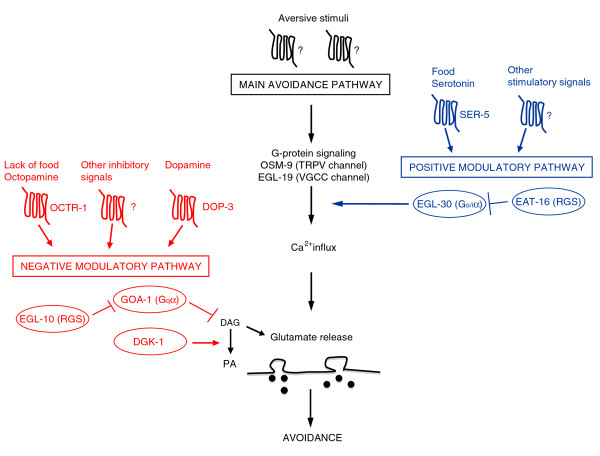
**A model for the ASH modulatory pathways**. The model is discussed in the text, in the Discussion section. The molecules in the ovals are those studied in this paper. Only the main players are depicted. The question marks indicate that only the type of molecule (GPCR) is known, while its precise identity is not. DAG, diacylglycerol; PA, phosphatidic acid. Arrows indicate proven activities or pathways. Stimuli and molecules upstream of the two modulatory pathways: food, dopamine, octopamine, serotonin, DOP-3, OCTR-1 and SER-5 have been identified by Wragg *et al. *[10], Harris *et al. *[11] and Ezak and Ferkey [35] as modulators of the response to dilute octanol.

A possible model for the way these proteins function in ASH is depicted in Figure 6. The model shows the main avoidance signalling pathway in which signals, triggered by different aversive stimuli, converge on the main signal transduction channel of ASH and OSM-9. Gating of OSM-9 generates a signal that is transmitted to the cell body and to downstream neurons to trigger avoidance responses. The model also depicts two opposing modulatory pathways. The negative one, with G_0/i_α GOA-1 as the key component, functions downstream of OSM-9 and inhibits neurotransmission by reducing DAG levels. EGL-10 acts in this pathway and affects avoidance by its established function, the inhibition of GOA-1 signalling, thus increasing the concentration of DAG levels at presynaptic sites. Its function is contrasted by the DAG kinase DGK-1 that inactivates DAG. In this model, on the basis of our results, the mechanisms of action of GOA-1 and EGL-10 in ASH appear to be largely the same as those at neuromuscular junctions in motoneurons. The positive modulatory pathway, with G_q_α EGL-30 as the key component, increases primary signalling as Ca^2+ ^transients are reduced in *egl-30 *mutants in response to high osmolarity (Figure 4a). The pathway functions upstream of OSM-9 and its effect on behaviour is contrasted by the RGS EAT-16. That EGL-30, in ASH, acts on signal generation and transduction is also supported by previous results showing that serotonin stimulation of the avoidance response to mechanical stimuli is mediated by an increase in ASH Ca^2+ ^transients [5]. Since serotonin modulates ASH avoidance responses through the SER-5 receptor and G_q_α signalling [11], the result is consistent with EGL-30 G_q_α acting upstream of OSM-9 gating. Thus the mechanism of action of EGL-30 in ASH appears to be different from that by which this protein acts at neuromuscular junctions, where it has been shown to act presynaptically in signal transmission by increasing DAG concentration via the phospholipase PLCβ EGL-8 and facilitating transmitter (acetylcholine) release [16,27,28]. Whether, in ASH, EGL-30 also acts presynaptically cannot be established on the basis of our experiments and will require further investigations. However, it is worth mentioning that the avoidance response of *egl-8 *mutants to the high osmotic strength stimulus was not reduced compared to that of wild-type animals (our unpublished observations) as would be expected on the basis of the neuromuscular junction mechanism. Similarly, our experiments are not sufficient to establish whether the RGS EAT-16, which inhibits the G_q_α positive regulatory pathway, acts in ASH on signal transduction or on transmission or on both.

We do not know the endogenous and/or environmental cue(s) to which the GOA-1 and EGL-30 modulatory pathways are responding, and they have not been investigated in this paper. The modulatory effects on ASH responses of the presence or absence of food and of serotonin, octopamine, dopamine and other transmitters have been described previously [5,8-10,12,34]. Important recent work has identified some of the ligands of the receptors and of the Gα proteins involved in the modulation of the response of ASH to dilute octanol. This response is modulated by the feeding status of the animal through the neurotransmitters serotonin and octopamine, the SER-5 and OCTR-1 receptors, respectively, and G_s_α (GSA-1), G_q_α (EGL-30) and G_0/i_α (GOA-1) signalling [9-11]. The response to dilute octanol has also been shown to be negatively modulated by dopamine [10], and DOP-3, a D2-like dopamine receptor, is necessary for this modulation. *dop-3 *mutants are hypersensitive to dilute octanol, and DOP-3 function is required in ASH as its expression in this neuron is sufficient to rescue the hypersensitivity of *dop-3 *mutants [35]. It has also been shown that, at least in cholinergic motoneurons, DOP-3 signals through GOA-1 to inhibit locomotion [36]. Together these data suggest that dopamine might contribute to the negative modulation of ASH through the DOP-3 receptor and the activation of GOA-1 signalling. The present study is focused on the role of EGL-10, GOA-1, EAT-16 and EGL-30 in the response of ASH neurons, not to dilute octanol, but to various aversive stimuli and in particular to high osmolarity. It is, however, reasonable to hypothesise that the neurotransmitters and/or neurohormones, as well as the receptors and the Gα signalling molecules, involved in the modulation of ASH are largely the same as those involved in the modulation of the response to dilute octanol.

Like the mammalian nociceptive neurons of the dorsal root ganglia, the *C. elegans *ASH sensory neurons detect stimuli of different nature (polymodality), use a TRPV channel as the main signal transduction channel and glutamate as a neurotransmitter. ASHs have thus proved to be among the most important models to study in live animals, with single-cell resolution, how nociceptive neurons function. The Gα and RGS molecules we have identified are largely conserved in evolution, and mammalian orthologs for each of them can be identified. Our results raise the possibility that also in mammals similar mechanisms might be in place to modulate the activity of nociceptive neurons in the pathway for pain sensation. The discovery of molecules involved in the modulation of signal transduction and signal transmission in nociceptor neurons and the elucidation of their mechanism of action may shed light on how pain is generated and how its control can go astray (that is, chronic pain) and may be useful for designing new pain control therapies.

## Conclusions

In *C. elegans*, the ASH sensory neurons have a central role in detecting aversive stimuli. The responsiveness of ASH to these stimuli is modulated by endogenous and/or environmental cue(s) that include the presence or absence of food and of serotonin, octopamine and dopamine. Two opposing signalling pathways mediate the effects of these cues. The RGS EGL-10 and the G_o/i_α protein GOA-1 are key elements of a negative modulatory pathway, while the RGS EAT-16 and the G_q_α protein EGL-30 function in a positive modulatory pathway. Within ASH, EGL-10 and GOA-1 act downstream of stimulus-evoked signal transduction and of the main transduction channel OSM-9. EGL-30 instead appears to act upstream by regulating Ca^2+ ^transients in response to aversive stimuli. EGL-10 and GOA-1 do not affect signal transduction and neuronal depolarization in response to aversive stimuli, but act in ASH presynaptically to modulate glutamate release in a fashion similar to that in which these two proteins modulate acetylcholine release at the neuromuscular junction.

## Methods

### Animals

Nematodes were grown and handled following standard procedures. Alleles and transgenic strains are described in the text. Wild-type animals were *C. elegans *variety Bristol strain N2. Alleles used in this work included *dgk-1*(*nu62*), *goa-1*(*n363*), *egl-30*(*n686*), *egl-10*(*md176*) and *eat-16*(*ad702*), and they were provided by the Caenorhabditis Genetics Center (CGC), which is funded by the National Institutes of Health (NIH) National Center for Research Resources (NCRR). The strain carrying the transgene for Ca^2+ ^imaging Ex[p*sra-6::G-CaMP*] and the strain carrying the transgene to test capsaicin avoidance Is[p*elt-2::gfp; *p*sra-6::TRPV1*] were kindly provided by C.I. Bargmann (Rockefeller University, New York, NY, USA). Transgenic strains used in this work were NA193 *gbEx506 *[p*sra-6::PTX; *p*elt-2::gfp*], NA842 *egl-10*(*md176*), *gbEx508 *[p*sra-6::egl-10; *p*R09E10.7::gfp*], NA273 *gbEx515 *[p*sra-6::eat-16; *p*elt-2::gfp*], NA879 *egl-10*(*md176*) and *gbEx516 *[p*odr-10::egl-10; *p*elt-2::gfp*]. Genetic crosses were used to obtain double mutants and to transfer transgenes to the appropriate genetic backgrounds. In all cases, the presence of the mutant alleles was verified by polymerase chain reaction (PCR) followed, when necessary, by sequencing. Worms were grown under uncrowded conditions at 20°C on NGM (Nematode Growth Medium) agar plates seeded with *Escherichia coli *strain OP50.

### Cell-specific expression constructs

Constructs for cell-specific expression of genes were obtained by PCR fusion as described by Hobert, 2002 [37] and included two steps. In the first step, sequences corresponding to the gene studied (genomic or cDNA) and to the chosen cell-specific promoter were amplified separately. In the second step, the cell-specific promoter and the gene fragment were fused by amplification using nested primers. Promoter sequences were amplified from genomic DNA and included sequences upstream as well as the ATG of the gene.

For the *sra-6 *promoter a 3 kb fragment was amplified using the following primers:

1. *sra-6 *forward = AGTGAGCATGAAGAAGGTAGAGGTTTTC

2. *sra-6 *reverse = GGCAAAATCTGAAATAATAAATATTAAATTCTGCG

3. *sra-6 *forward nested = CATGTTAGATAGTATGCTGCACTATAAGG

For the *odr-10 *promoter a 7 kb fragment was amplified using the following primers:

1. *odr-10 *forward = GGGACGTGCGAAATAGCATTGG

2. *odr-10 *reverse = AGCTGTAAGGTATCTTAATG

3. *odr-10 *forward nested = GATATCTACTTAAATATATAGGGACGTGCG

Because these promoters were used to drive transcription of different target genes, the reverse series of primers had, at the 5' end, 25 additional nucleotides complementary to the 5' extremities of the amplified gene fragment to which they had to be fused.

*egl-10 *cDNA (3 kb) was amplified from plasmid pUT35A (a gift from M. Koelle, Yale University, New Haven, CT, USA). In this plasmid, the *egl-10 *cDNA was fused to the 3' UTR of the *unc-54 *gene, and for the fusion the same reverse primer as that used for the first step was used. Primers were as follows:

1. *egl-10 *forward = ATGGCTCTACCAAGATTGAGGGTAAATG

2. *egl-10 *reverse = GGAAACAGTTATGTTTGGTATATTGGG

*ptx *cDNA (1.5 kb) was amplified from plasmid pJT40 (a gift from M. Koelle, Yale University, New Haven, CT, USA). Primers were as follows:

1. *ptx *forward = ATGGACGATCCTCCCGCCACC

2. *ptx *reverse = GCCGACTAGTAGGAAACAGT

3. *ptx *reverse nested = CAGTTATGTTTGGTATATTGG

*eat-16 *coding sequence (2.4 kb) was amplified from genomic DNA. Primers were as follows:

1. *eat-16 *forward = ATGATGCCACCGTTGACCAAG

2. *eat-16 *reverse = ATTGAACATCAACGCCTACA

3. *eat-16 *reverse nested = TTATGTAACAACTCCGGTTCTG

### Transgenic nematodes

Germline transformation was performed as described previously [38]. The coinjection marker used was p*elt-2*::GFP (pJM67, a gift from J. McGhee, Calgary, AB, Canada), which drives green fluorescent protein (GFP) expression in intestinal cells, and p*R09E10.7*::GFP, which drives GFP expression in the pharynx. The expression constructs were all injected at 50 ng/μl together with pJM67 (20 ng/μl) and p*R09E10.7*::GFP (50 ng/μl). At least three independent lines for each construct were tested for the avoidance phenotype (see Additional file 2).

### Behavioural assays

Avoidance of high osmotic strength, quinine and copper ions was assayed using the drop test [6] on NGM agar plates 6 cm in diameter. A single drop of repellent (1 M glycerol, 10 mM quinine, 1 mM copper) was placed near the tail of an animal moving forward. The animal started a backward motion when, by capillary action, the repellent reached the tip of its mouth, where sensory cilia were exposed. The response was scored as positive if the animal backed up within 3 seconds. The results are expressed as avoidance index (AI), which is the number of positive responses divided by the total number of trials. Delay in avoidance response was determined using the dry drop test [6,12]. In brief, a well-fed young adult animal was transferred to an unseeded agar plate and allowed to recover for at least 2 minutes. A small drop of repellent (1 M glycerol or 50 μM capsaicin) was then placed in the path of the worm as it moved forward. The time interval in seconds (delay) between the initial contact of the animal with the solution and the response (backward movement) was recorded. In Table 1, only animals responding within 7 seconds were used to calculate the delay time. Octanol (100%) avoidance was tested as described previously [8] and is expressed as the number of seconds that animals took to start backward movement. The response to anterior light body touch was tested as described previously [22], and the results are reported as avoidance indexes. For all of the avoidance assays (osmotic strength, quinine, copper ions, anterior light body touch, octanol and delay in avoidance), at least 50 animals of each genetic makeup were tested in groups of 10 or 20 with three drops/animal.

The frequency of spontaneous reversals was measured as described previously [30] with some modifications. A single, well-fed, young adult animal was placed on a standard unseeded NGM agar plate. After a brief equilibration time of 3-5 minutes, the movement of the animal was observed for 3 minutes and the number of times the animal stopped and reversed its forward movement was counted. At least 50 animals were tested, and the results are reported as reversals/minute.

### *In vivo *Ca^2+ ^imaging of ASH neurons

Ca^2+ ^imaging followed the method described previously [24] with some modifications. Well-fed, young adult hermaphrodites carrying the *sra-6::G-CaMP *transgene were picked under a fluorescence stereoscope. To minimize signal variations due to G-CaMP expression levels, only animals with similar expression were used. Animals were immediately glued with 2-octyl cyanoacrylate adhesive onto a chilled, hydrated 2% agarose pad on a glass coverslip. The coverslip was attached with silicone to a flow chamber (RC-26GLP; Warner Instruments, Hamden, CT, USA) perfused with saline at a rate of 1.0 ml/minute. Glycerol, quinine and copper were dissolved in saline buffer to final concentrations of 1 M, 10 mM and 1 mM, respectively, and delivered under light hydrostatic pressure through a glass needle near the tip of the animal's head. The movement of the needle was controlled through a manual micromanipulator. Each imaging trial lasted for approximately 40 seconds with the following temporal sequence: approximately 10 seconds baseline, approximately 3 seconds stimulation and approximately 35 seconds recovery. Animals were stimulated for a maximum of three trials with an intertrial interval of 3-5 minutes if they stayed healthy. Optical recordings were taken with a Leica TCS SP2 confocal microscope (Leica Microsystems, Wetzlar, Germany). Fluorescence images were acquired (five frames/second), and a region of interest (ROI) with ASH cell body in focus was chosen. Quantitative measures of the intensity of the G-CaMP fluorescence from the ROI were obtained from the photomultiplier data. Changes in fluorescence intensity over time were calculated relative to averaged prestimulus baseline, ΔF/F. Both incremental ratios over time and the maximal incremental ratios from each trial were calculated. The incremental ratios were calculated for every frame (five points/second). Trials in which artifacts such as movement of the animal's head or if strong bleaching was conspicuous were discarded.

### Statistical analysis

Means, standard deviations and standard errors of mean values were calculated for each data set. The statistical significance was determined using Student's *t*-test comparing each data set against the control.

## Authors' contributions

GE conceived and designed most of the experiments, carried out the molecular genetic manipulations and the behavioural assays and drafted the manuscript. MRA performed behavioural assays and the Ca^2+ ^sensing experiments. CB did the initial characterization of the avoidance responses of the mutants. EDS participated in the design of the experiments and the genetic manipulations, performed behavioural assays and helped in the writing of the manuscript. PB conceived the study, participated in its design and coordination and helped in writing the manuscript. All authors read and approved the final manuscript.

## Supplementary Material

Additional file 1 **Supplementary Table 1**. Avoidance response to buffer. Click here for file

Additional file 2 **Supplementary Table 2**. Avoidance response to 1 M glycerol of independent transgenic lines. Click here for file
