# Supplementary material for: The G Protein regulators EGL-10 and EAT-16, the Giα GOA-1 and the Gqα EGL-30 modulate the response of the C. elegans ASH polymodal nociceptive sensory neurons to repellents
Source: BMC Biol. 2010 Nov 11;8:138. doi: 10.1186/1741-7007-8-138 (PMC2996360; doi:10.1186/1741-7007-8-138)
Supplement: Additional file 1 — Supplementary Table 1. Avoidance response to buffer. [file 1741-7007-8-138-S1.DOC]

**Supplementary Table 1. Avoidance response to buffer**

| **Genotype** | **Avoidance index (Mean ± SEM)** | **N** |
| --- | --- | --- |
| N2 | 0.21 ± 0.01 | 50 |
| *eat-16* | 0.20 ± 0.02 | 50 |
| *psra-6::PTX* | 0.19 ± 0.01 | 50 |
| *dgk-1* | 0.33 ± 0.01 | 40 |
| *egl-10;eat-16* | 0.21 ± 0.02 | 40 |
| *egl-10;goa-1* | 0.20 ± 0.02 | 40 |
| *egl-30;eat-16* | 0.18 ± 0.01 | 40 |
| *egl-30;psra-6::PTX* | 0.22 ± 0.02 | 40 |
