# Supplementary material for: The G Protein regulators EGL-10 and EAT-16, the Giα GOA-1 and the Gqα EGL-30 modulate the response of the C. elegans ASH polymodal nociceptive sensory neurons to repellents
Source: BMC Biol. 2010 Nov 11;8:138. doi: 10.1186/1741-7007-8-138 (PMC2996360; doi:10.1186/1741-7007-8-138)
Supplement: Additional file 2 — Supplementary Table 2. Avoidance response to 1 M glycerol of independent transgenic lines. [file 1741-7007-8-138-S2.DOC]

**Supplementary Table 2. Avoidance response to Glycerol 1 M of independent transgenic lines**

| **Genotype** | **Avoidance index (Mean ± SEM)** | **N** |
| --- | --- | --- |
| *egl-10;psra-6::egl-10* #1 | 0.60 ± 0.01 | 50 |
| *egl-10;psra-6::egl-10* #2 | 0.65 ± 0.02 | 50 |
| *egl-10;psra-6::egl-10* #3 | 0.64 ± 0.01 | 50 |
| *egl-10;podr-10::egl-10* #1 | 0.38 ± 0.01 | 40 |
| *egl-10;podr-10::egl-10* #2 | 0.36 ± 0.02 | 40 |
| *egl-10;podr-10::egl-10* #3 | 0.40 ± 0.02 | 40 |
| *psra-6::PTX* #1 | 0.84 ± 0.01 | 40 |
| *psra-6::PTX* #2 | 0.87 ± 0.02 | 40 |
| *psra-6::PTX* #3 | 0.85 ± 0.02 | 40 |
| *egl-10;psra-6::PTX* #1 | 0.78 ± 0.01 | 50 |
| *egl-10;psra-6::PTX* #2 | 0.83 ± 0.02 | 50 |
| *egl-10;psra-6::PTX* #3 | 0.82 ± 0.01 | 50 |
| *egl-10;eat-16;psra-6::eat-16* #1 | 0.42 ± 0.02 | 40 |
| *egl-10;eat-16;psra-6::eat-16* #2 | 0.38 ± 0.01 | 40 |
| *egl-10;eat-16;psra-6::eat-16* #3 | 0.43 ± 0.01 | 40 |
| *egl-30;psra-6::PTX* #1 | 0.70 ± 0.01 | 50 |
| *egl-30;psra-6::PTX* #2 | 0.68 ± 0.02 | 50 |
| *egl-30;psra-6::PTX* #3 | 0.72 ± 0.01 | 50 |
